# Chemical fingerprinting, antimicrobial, antioxidant, anti‐inflammatory, and anticancer potential of greenly synthesized silver nanoparticles from pistachio (*Pistacia vera*) nuts and senna (*Cassia angustifolia Vahl*.) leaves

**DOI:** 10.1002/fsn3.4148

**Published:** 2024-04-30

**Authors:** Saba Irshad, Sabahat Iftikhar, Muhammad Riaz, Azra Mahmood, Afaq Mushtaq, Yasar Saleem, Rahat Shamim, Quzi Sharmin Akter

**Affiliations:** ^1^ School of Biochemistry and Biotechnology University of the Punjab Lahore Pakistan; ^2^ Department of Allied Health Sciences University of Sargodha Sargodha Pakistan; ^3^ Centre for Excellence in Molecular Biology University of the Punjab Lahore Pakistan; ^4^ Food and Biotechnology Research Centre, PCSIR Labs Complex Lahore Pakistan; ^5^ Punjab University College of Pharmacy (PUCP) University of the Punjab Lahore Pakistan; ^6^ Department of Genetics and Animal Breeding, Faculty of Animal Science and Veterinary Medicine Patuakhali Science and Technology University Patuakhali Bangladesh

**Keywords:** anticancer, anti‐inflammatory, *Cassia angustifolia*, *Pistacia vera*, *silver nanoparticles*

## Abstract

There is a growing interest in standardizing the biocompatible, cost‐effective, and eco‐friendly manufacturing techniques for metallic nanostructures due to their widespread applications in the industrial and medical sectors. In recent decades, green synthesis has been proven as the most suitable technique for synthesizing metal nanoparticles. The present research study investigates the use of *Cassia angustifolia* (senna) leaves and *Pistacia vera* (Pistachio) nuts to prepare crude aqueous extracts, ethanolic extracts, and biogenic silver nanoparticles (AgNPs). The prepared aqueous extracts were used as reducing, stabilizing, and capping agents for the production of silver nanoparticles. These AgNPs were characterized by scanning electron microscopy (SEM), Fourier‐transform infrared spectroscopy (FTIR), and ultraviolet–visible (UV–Vis) spectroscopy. The outcomes validated the formation of stable AgNPs with bioactive functional components. In vitro antibacterial, anticancer, anti‐inflammatory, and antioxidant potentials were assessed by Kirby–Bauer disk diffusion test, MIC test, MBC test, MTT assay, BSA denaturation inhibition assay, and DPPH antioxidant assay, respectively. Results confirmed that the tested plant extract possesses a variety of bioactive compounds with various biological activities and is therapeutically effective. These findings verified that *C. angustifolia* and *P. vera* are promising bioresources for the synthesis of therapeutic extracts and nanostructures with commendable therapeutic potency.

## Graphical abstract

In recent decades, green synthesis has been attested as the most suitable technique for synthesizing metal nanoparticles. *C. angustifolia* and *P. vera* extract possess a variety of bioactive compounds with various biological activities and are therapeutically effective. *C. angustifolia* and *P. vera* are promising bioresources for the synthesis of therapeutic extracts and nanostructures with commendable therapeutic potency.

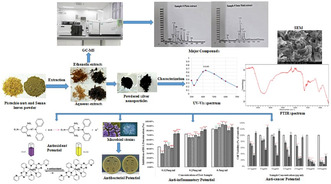

## 1 INTRODUCTION

Since prehistoric times, medicinal plants have been utilized in traditional preventive and curative therapeutics due to their ability to naturally synthesize chemical compounds with antiviral, antibacterial, antipathogenic, antioxidant, antiaging, analgesic, antitumor, and anti‐inflammatory properties. These bioactive chemical constituents of plants are known as phytonutrients or phytochemicals (Molyneux et al., 2007). Plants produce phytochemicals as pharmacologically active compounds for their own protection and survival, either through primary or secondary metabolism. Numerous research studies have revealed the bioactivity of phytochemicals in mitigating oxidative stress (Upadhyay & Dixit, 2015), boosting immunity, preventing hemolysis (Dubey et al., 2017), maintaining blood glucose levels (Yin et al., 2008), sustaining blood pressure, decreasing the risks of cardiovascular diseases (Krga & Milenkovic, 2019), preventing oxidative stress and inflammation (Serafini & Peluso, 2016), wound healing (Thangapazham et al., 2016), and assessing humans with carminative, antimicrobial, anti‐inflammatory, antioxidation, antispasmodic, antiallergic, antiaging, anticancer, neuroprotective, hepatoprotective, hypolipidemic, hypotensive, immunomodulatory, and analgesic benefits (Gupta & Prakash, 2014).

Besides medicinal plants, the extraordinary advancement in nanotechnology has resulted in the widespread use of nanomaterials in biomedical research, cosmetics, healthcare, disease diagnosis, targeted drug and gene delivery, and the feed and food industry (Amaladhas et al., 2012; Bakhshi & Bagherzade, 2021; Mabrouk et al., 2021). The traditional physical and chemical methods of nanomaterial synthesis had several drawbacks, such as low production rates, high cost of manufacturing, usage of hazardous chemicals, toxic by‐product formation, huge energy requirements, and contamination from precursor compounds (Osman et al., 2024; Thakkar et al., 2010). The biosynthesis of nanoparticles is ascertained as a cost‐effective, efficient, eco‐friendly, easy to scale‐up, renewable, and sustainable alternative to the physical and chemical approaches (Rafique et al., 2017).

Plant‐derived nanoparticles are thought to be the most bioactive due to the presence of phytonutrients including terpenoids, alkaloids, limonoids, isoflavonoids, flavonoids, carotenoids, ω‐3 fatty acids, polyphenols, phytosterols, glucosinolates, anthocyanins, vitamins, probiotics, and phytohormones (Gupta & Prakash, 2014). These biomolecules act as reducing, capping, and stabilizing agents (Bindhu et al., 2013). The abundance of phytocompounds in medicinal plants makes them an excellent source for large‐scale nanomaterial synthesis.

*P. vera* and *C. angustifolia* belong to the rosids clade of the angiosperms. *C. angustifolia* is a prominent herb utilized in the Ayurvedic, Unani, and Allopathic medicinal systems (Amaladhas et al., 2012). It is traded under the name ‘Sana Makkahi’. *C. angustifolia* is historically quite significant as Prophet Muhammad (P.B.U.H.) pioneered its usage in herbal medicines in the blessed city of Mecca (Sultana et al., 2012). *C. angustifolia* contains sennosides, anthraquinones, flavonoids, naphthalene derivatives, tannins, phytosterols, essential oils (Abbas & Rani, 2020), anthrones, palmidin A, rhein, resin, mucin, and emodin (Laghari et al., 2011). It is utilized to treat digestive diseases, menstrual problems, skin diseases, respiratory disorders, heart diseases, epilepsy (Sultana et al., 2012), rheumatism, anemia, jaundice, typhoid, leukoderma, leprosy, hepatomegaly, piles, migraine, asthma, obesity, constipation, and cough (Balasankar et al., 2013). It also possesses antioxidant, anti‐inflammatory, antifungal, and anticancer potential (Osman et al., 2017). In Pakistan, *C. angustifolia* leaves were believed to prevent and treat SARS‐CoV‐2 infection. However, no research study has verified the effectiveness of any bioactive compound, extract, or herb against this virus (Alghamdi, 2021).

*P. vera* is a small, deciduous plant of Anacardiaceae family native to the Central Asia and Middle East. It is mainly found in Iran, Turkey, Syria, Italy, Greece, and the USA (Saitta et al., 2014). *P. vera* bears fruit in the form of edible nuts. This fruit is classified as a drupe. Raw pistachios are a rich source of antioxidants, dietary fibers, minerals, vitamins, phytominerals, unsaturated fatty acids, protein, and phytochemicals like γ‐tocopherol, anthocyanins, polyphenols, cardanols, phytosterols, xanthophyll carotenoids, and resveratrol (Bulló et al., 2015). They are known to regulate blood pressure and blood glucose, control weight, reduce triglyceride and cholesterol levels, modulate gut microbiota (Hernández‐Alonso et al., 2016), decrease risks of cardiovascular ailments or neurodegenerative disorders (Dreher, 2012), prevent chronic eye diseases, improve bone density, enhance blood vessels flexibility, and possess chemopreventive characteristics (Seifaddinipour et al., 2018).

The present research study is devised to prepare ethanolic extracts, aqueous extracts, and silver nanoparticles utilizing *P. vera* (Pistachio) nuts and *C. angustifolia Vahl*. (Sana Makki) leaves. The purpose is to ascertain antimicrobial, antioxidant, anti‐inflammatory, and anticancer potential of the prepared plant‐based extracts and nanoparticles. This study aims to develop an understanding of the use of medicinal plants as cost‐effective, eco‐friendly, bio‐renewable, sustainable, and diversified resources for the preparation of herbal therapeutics with pharmacologically active components that may have widespread applications.

## 2 MATERIALS AND METHODS

### 2.1 Collection and processing of source plants

*Pistacia vera* nuts and *Cassia angustifolia* leaves were acquired from a local market in Lahore, Pakistan. These plant materials were identified and authenticated by Dr. Abdul Rehman Khan Niazi from the Institute of Botany, University of the Punjab. Pistachios were dehulled, sliced into bits, and blended into a fine powder. Their skin was not removed. The powdered pistachios and senna leaves were then stored separately in air‐tight containers.

### 2.2 Preparation of crude extracts

Dried senna leaves and pistachios powders (30 g each) were weighed and immersed in separate Erlenmeyer flasks containing 300 mL of distilled water. The mixtures were heated at 60°C for 30 min with intermittent stirring and then set aside to cool. They were then filtered, and the resulting filtrates were stored at 4°C.

Ethanolic extracts were prepared using the conventional cold maceration method. Thirty grams of dried plant powders was weighed and soaked in 150 mL of 96% standard‐grade ethanol. The mixtures were incubated at 37°C and 120 rpm for 72 h in an incubator with continuous shaking. They were then filtered and the filtrates were refrigerated at 4°C for future use.

### 2.3 GC–MS (Gas chromatography–mass spectrometry) analysis

The GC–MS analysis of ethanolic extracts was conducted with an Agilent Technologies 6890 Series gas chromatograph coupled with (an Agilent) 5973 Mass Selective detector and driven by Agilent chemstation software. A DB‐5MS capillary column was used (30 m × 0.25 mm internal diameter, 0.25 μm film thickness). Ultra‐pure helium was used as the carrier gas at 1.0 mL/min flow rate and 37 cm/s linear velocity.

The injector temperature was set at 220°C with the initial oven temperature at 60°C and holding time of 4 min which was programmed to increase the temperature up to 260°C at the rate of 10°C/min.

Injections of 0.2 μL were made in the splitless mode. The mass spectrometer was operated in the electron ionization mode at 70 eV. Other MS operating parameters were as follows: ion source temperature of 230, 150°C quadruple temperature, 4‐min solvent delay, and 50–700 amu scan range.

### 2.4 Lyophilization of extracts

The prepared aqueous extracts were partitioned into two portions: one half was kept as a stock solution for nanoparticle formation, while the second half was lyophilized through freeze drying under low pressure to obtain the dried concentrates for performing assays. The ethanolic extracts were not utilized for the formation of nanoparticles, so, they were entirely lyophilized.

### 2.5 Green synthesis of silver nanoparticles (AgNPs)

The 100 mL of 10 mM aqueous silver nitrate (AgNO_3_) solution was heated for 15 min at 60°C in separate Erlenmeyer flasks. Then, aqueous plant extracts (50 mL) were added into the flasks dropwise under continuous magnetic stirring. Using 1.0 M NaOH solution, the pH of the reaction mixtures was adjusted to pH = 8.0. The addition of extract changed the color of the mixtures from colorless to dark ash brown with precipitates due to SPR (surface plasmon resonance), indicating the AgNP formation. These nanoparticle suspensions were centrifuged for 20 min at 4°C and 4000 *g*. The supernatant was discarded, and the pellets were resuspended in autoclaved water and centrifuged thrice to remove the excess residues adsorbed on the surface of AgNPs. The purified pellets were dried and stored.

### 2.6 Characterization of nanoparticles

The optical absorbance was determined by ultraviolet–visible spectrum analysis performed at 200–700 nm on a SHIMADZU UV‐1700 Pharmaspec Spectrophotometer. The FTIR (Fourier‐transform infrared spectroscopy) spectrum was recorded for the structural characterization. Scanning electron micrograph images were taken on FEI Nova NanoSEM 450 operated at 10.00 kV.

### 2.7 Antibacterial activity

The antibacterial activity of test samples was evaluated using preidentified *Staphylococcus aureus* (Gram‐positive) and *Escherichia coli* (Gram‐negative) strains acquired from the Services Hospital, Lahore, Pakistan. The aqueous solutions of *P. vera* and *C. angustifolia* AgNPs (1 mg/mL each) were prepared by ultrasonication utilizing 20 kHz of ultrasonic rates/frequencies. Then, 0.001 g/mL solutions of the lyophilized ethanolic and aqueous extracts were prepared in ethanol and distilled water, respectively.

### 2.8 Kirby–Bauer disk diffusion test

Antibiotic susceptibility of test samples was measured using the method described by Balouiri et al. (2016). MHA (Muller–Hinton Agar) was prepared and poured onto appropriately labeled Petri dishes. Following its solidification, a single bacterial suspension (0.5 McFarland) of either *E. coli* or *S. aureus* was inoculated into each plate with cotton swabs. The filter‐paper disks (size = 5 mm) were then soaked in the test samples and mounted on the respective agar plates at an adequate distance.

Ampicillin (1 g/10 mL concentration) and distilled water were used as positive and negative controls, respectively, while ethanol was utilized for the accurate assessment of antibacterial action of ethanolic extracts because of the toxic effect of alcohol on bacterial cells. All of the Petri plates were incubated for 24 h at 37°C in an incubator. The diameter of the zone of inhibition (ZOI) was measured to assess the antibacterial potential of test samples. It included the disk diameter as well. The ethanol's ZOI value was deducted from that of ethanolic extracts. All the experiments were performed in triplicate.

### 2.9 Determination of minimum inhibitory concentration

MIC was measured using the broth dilution method described by Balouiri et al. (2016). The assay was carried out in 96‐well plates using MHB (Mueller–Hinton Broth). Direct colony suspension was done to make 0.5 McFarland microbial inoculum. Sterilized MHB (50 μL) was added from well 1 to well 12. In well 2, test sample (100 μL) was transferred and mixed with broth. From well 2 to well 11, a two‐fold microdilution was performed. Fifty microliters of ampicillin (well 1) was used as a positive control. MHB (well‐12) was the negative control. The microplates were then incubated at 37°C for 24 h.

Following incubation, the microplates were examined for bacterial growth. The lowest test samples' concentration that inhibited the bacterial growth was considered the MIC.

### 2.10 Determination of minimum bactericidal concentration

Sterile MHA‐containing Petri plates were prepared. Broth dilutions of test samples that did not demonstrate apparent bacterial growth during MIC test were subcultured in their respective plates using a sterilized inoculating loop. These plates were incubated at 37°C and observed for the presence of bacterial growth after 24 h.

### 2.11 Screening of anti‐inflammatory activity by inhibition of BSA (bovine serum albumin) denaturation assay

The inhibition of BSA denaturation assay was performed using a modified version of the method described by Bailey‐Shaw et al. slightly modified by Abbas and Rani (2020). In this method, 450 μL of 5% aqueous solution of BSA was homogenized with 50 μL of test samples at varied concentrations (0.125, 0.25, and 0.5 mg/mL). The reaction mixtures were incubated at 37°C for 15 min before being heated in a water bath at 70°C for 10 min followed by cooling at room temperature. Then, 2.5 mL of PBS (phosphate‐buffered saline) with pH = 7.2 was added to each mixture. The control was made by thoroughly mixing PBS (2500 μL), BSA (450 μL), and distilled water (50 μL). Diclofenac sodium in three varying concentrations (0.125 mg/mL, 0.25 mg/mL, and 0.5 mg/mL) was utilized as the standard. The test samples served as blanks. Each sample's absorbance was measured with a ultraviolet–visible spectrophotometer at 660 nm. This assay was carried out in triplicate.

The following formula was used to determine the percent inhibition of albumin denaturation:

$$\%\text{Denaturation inhibition}=\left\{\left(\text{Control}\;\mathrm{OD}-\text{Sample}\;\mathrm{OD}\right)/\text{Control}\;\mathrm{OD}\right\}\times 100.$$

### 2.12 In vitro DPPH (2,2‐diphenyl‐1‐picrylhydrazyl) antioxidant assay

The DPPH antioxidant assay was performed using a modified version of the method described by Riaz et al. (2017). 0.5‐mL aliquots of the test samples at three concentrations (0.125, 0.25, and 0.5 mg/mL) were prepared in 3‐mL ethanol (standard grade). Then, 0.004% (w/v) DPPH ethanol solution (0.5 mL) was added to each aliquot. The mixtures were then vortexed and left in the dark at room temperature for 30 min. Meanwhile, the aliquot's color changed indicating the purple DPPH reduction. Ascorbic acid was employed as a reference. Each sample's absorbance was measured at 517 nm using a ultraviolet–visible spectrophotometer against a blank of DPPH ethanol solution.

Free radical scavenging activity of test samples was calculated by using the following formula:

$$\text{DPPH scavenging effect}\;\left(\%\right)=\left\{\left(A_{c}-A_{t}\right)/A_{c}\right\}\times 100.$$

*A*_t_ and *A*_c_ represent the absorbance of the test sample and control, respectively.

### 2.13 MTT (3‐{4, 5‐dimethylthiazol‐2‐yl}‐2,5 diphenyl tetrazolium bromide) anticancer assay

#### 2.13.1 Preparation of test samples

The phytoextracts were dissolved in DMSO and then filtered with the help of regenerative cellulose filter to avoid contamination. AgNPs were dissolved in water using sonicator and sterilized with the help of ultraviolet radiations. Seven different concentrations (18.5, 37, 65, 125, 250, 500, and 1000 μg/mL) of each test sample were made in high glucose DMEM as per by specifications of the experiment.

#### 2.13.2 Cell revival

HepG2 cell line was used in this study. Cells were revived in high glucose DMEM by first thawing the cryovial, then adding it into 2 mL of FBS (fetal bovine serum) and 8–10 mL of high glucose DMEM. After centrifugation at 1000 *g* for 8 min, the pellet was plated in 75‐mL cell culture flask and incubated for 24 h.

#### 2.13.3 Cell plating and treatment

2 × 10^4^ cells were plated in 24 wells (seven doses, one control, each in triplicate) of the 96‐well plates (each for one test sample). After 24 h of incubation, different doses of test samples were added to the cells in each plate as in the order of: Control, 18.5, 37, 65, 125, 250, 500, and 1000 μg/mL.

Next day, MTT assay was performed in which 100 μL of MTT solution (5 mg/mL in 1X PBS) was transferred to each well after washing with PBS. The microplates were wrapped under aluminum foil and kept in the incubator at 37°C for 3 h. After incubation, the media were aspirated carefully so that no Formazan crystals were removed from each well. Then, 100 μL of DMSO was added to each well. The 96‐well plates were shaken for 10 s so that the Formazan crystals were dissolved resulting in the purple‐colored solution. Spectrostar Nano (BMG LABTECH) microplate reader was used to measure the optical density at 570 nm and 630 nm (Zughaibi et al., 2022).

### 2.14 Statistical analysis

All the invitro evaluation tests were performed in triplicate and the results were presented as means ± SD. For statistical comparisons, results were analyzed by one‐way ANOVA followed by Dunnett's multiple comparisons test using IBM® SPSS® Statistics software. The cell viability during MTT assay was evaluated using GraphPad Prism Version 8.0 software. Statistical differences were considered significant at *p* < .05.

## 3 RESULTS AND DISCUSSION

Dehulling, chopping, and blending the pistachios yielded a pale yellow‐green colored powder. The prepared ethanolic extract of pistachio was yellow, oily‐textured liquid while the aqueous solution was light‐green colored with visible oily phase. The purchased senna leaves powder was dark green. The ethanolic solution of senna leaves was dark‐brown and the aqueous filtrate was reddish‐brown.

**FIGURE 1:**
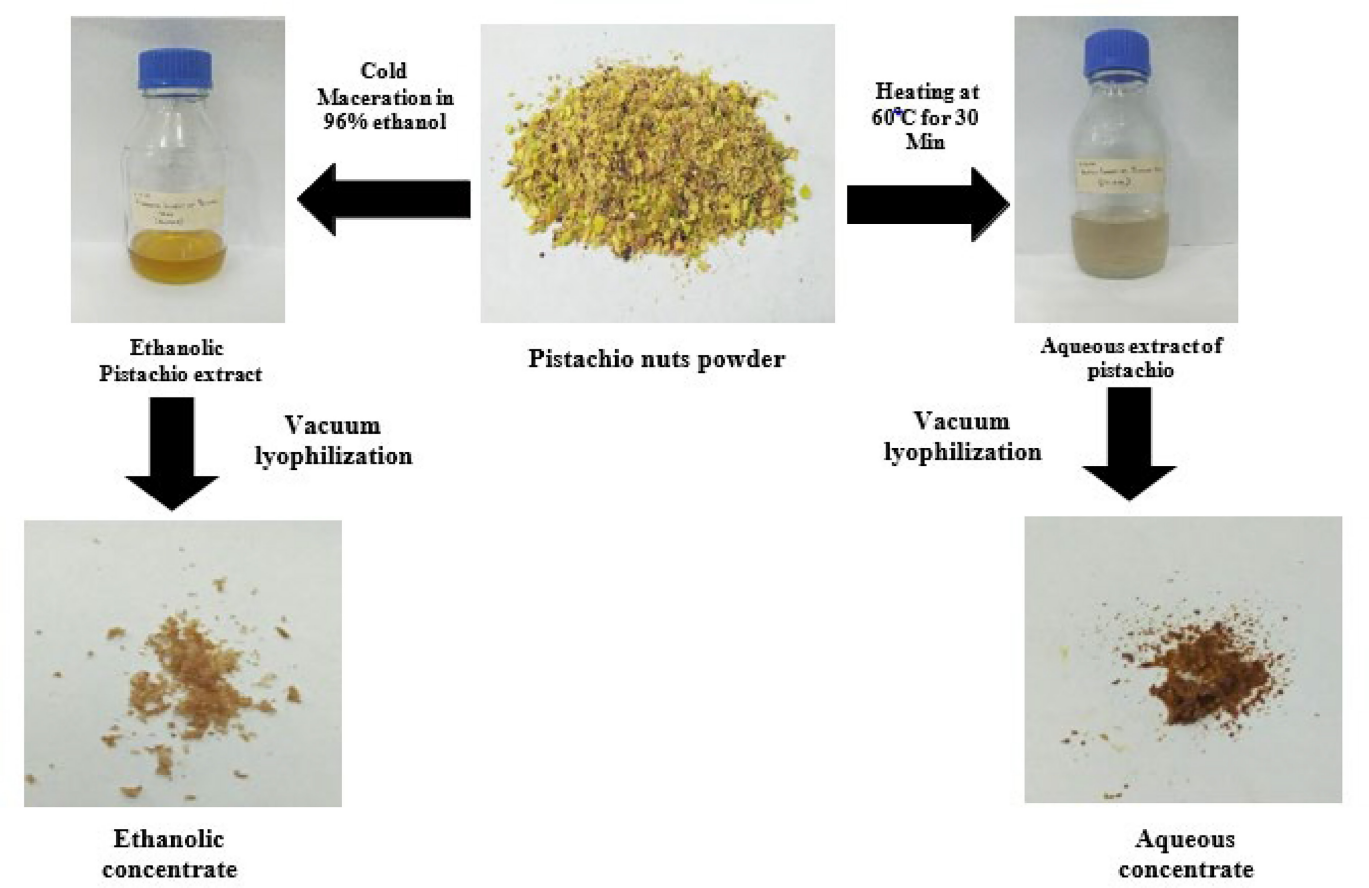
Preparation of aqueous and ethanolic extracts from pistachio.

These extracts were then lyophilized using vacuum lyophilizer. The aqueous pistachio extract became a light brown powder following lyophilization, whereas the ethanolic concentrate was dark brown (Figure 1). The yields of aqueous and ethanolic extraction of pistachio nuts were 3.6 and 2.8 g per 30 g, respectively.

**FIGURE 2:**
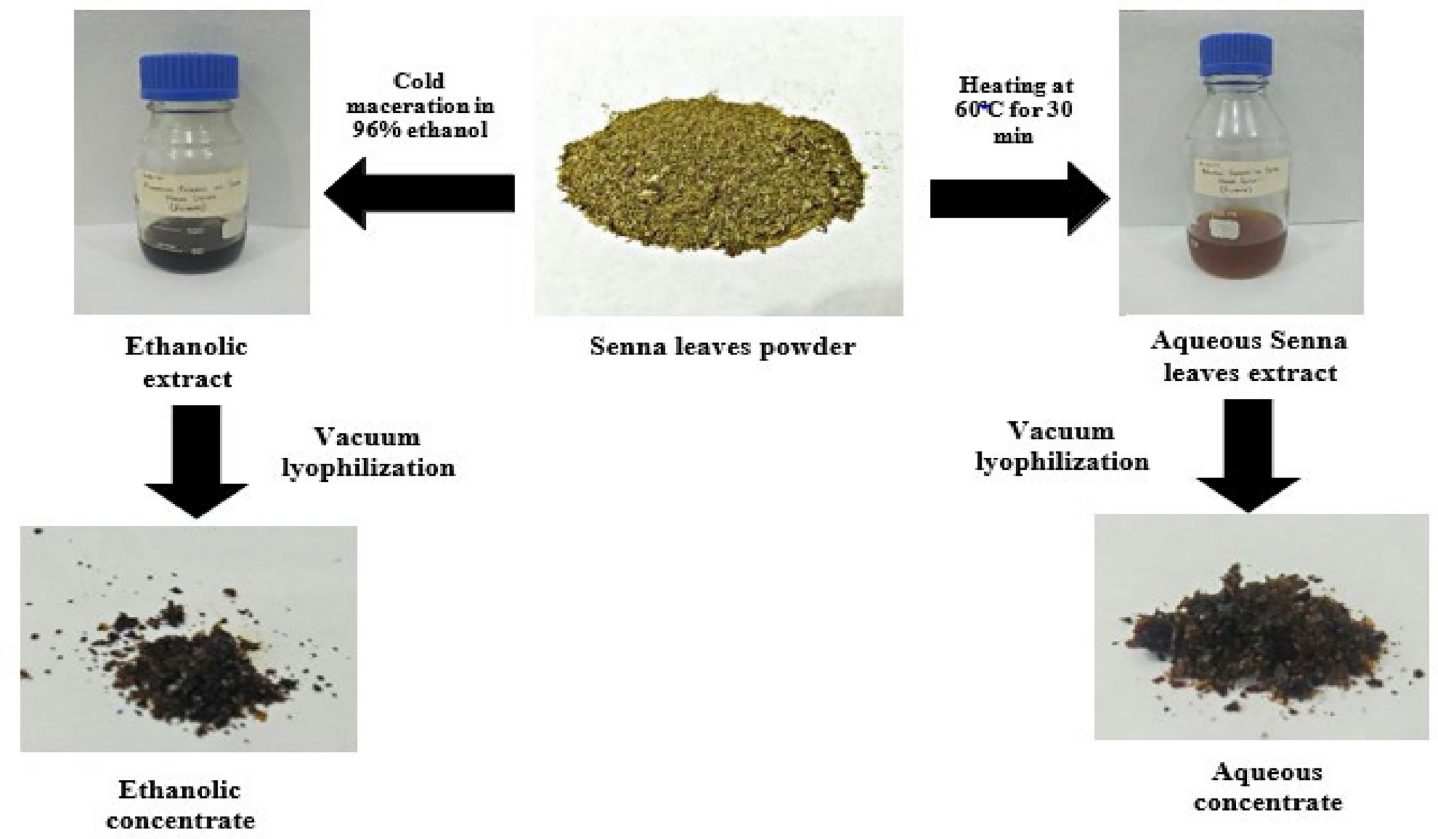
Preparation of senna‐based crude extract.

The ethanolic senna leaves extract turned into a jet black powder and the aqueous senna leaf concentrate acquired dark brown/black in hue (Figure 2). The yields of aqueous and ethanolic extraction of senna leaves were 3.76 and 3.0 g per 30 g, respectively.

### 3.1 GC–MS analysis of the ethanolic extracts

**FIGURE 3:**
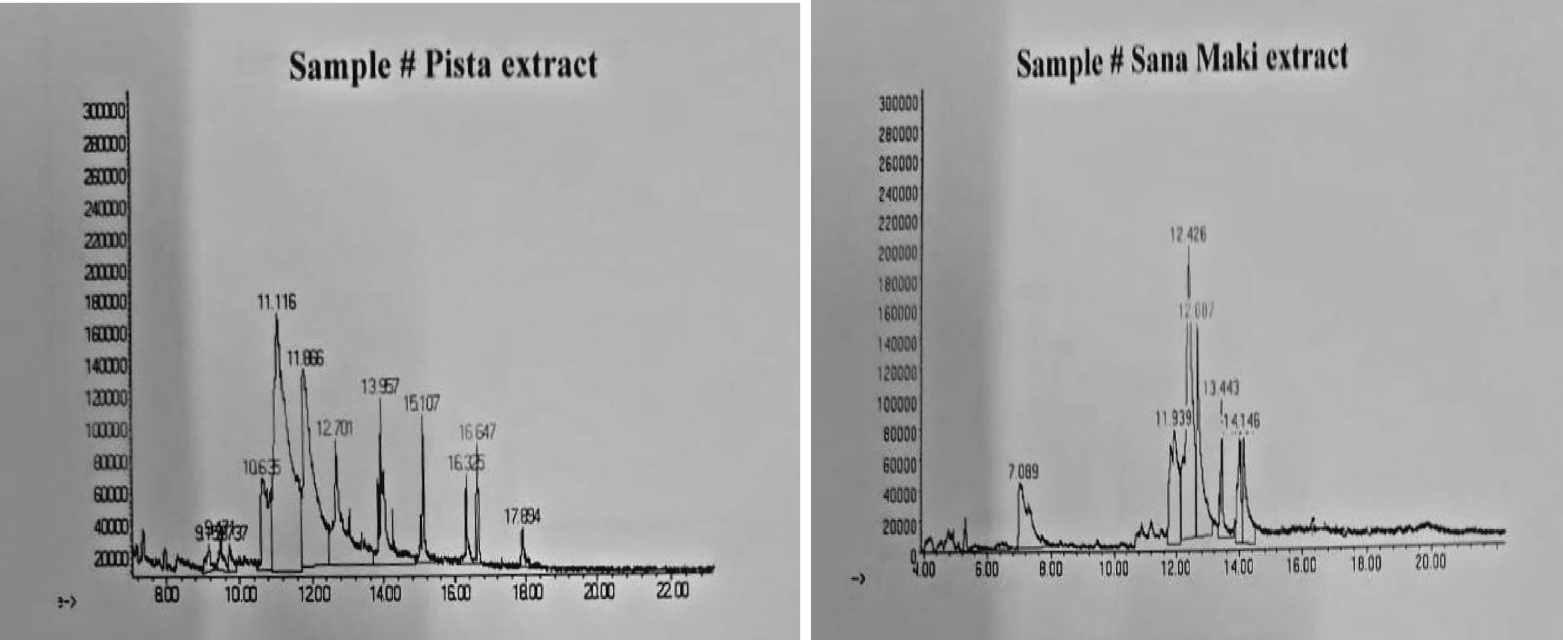
GC–MS chromatogram of ethanolic extracts of pistachio nuts and senna leaves.

GC–MS analysis of ethanolic pistachio nuts and senna leaf extracts identified the presence of major compounds with their respective percent area and retention time as shown by the presence of various peaks in the chromatogram (Figure 3). The compounds were identified by direct comparison of the retention times, mass spectral data, and fragmentation pattern with those in the National Institute of Standards and Technology (NIST) library (Tables 1 and 2).

**TABLE 1:** Phytochemicals identified in ethanolic extract of *Pistacia vera* nuts through GC–MS analysis.

Sr. No	Retention time	Compound name	Function	Area%
1	9.158	Nonadecane	Volatile oil component, Plant metabolite (Mojarrab et al., 2013)	1.13
2	9.471	Phenol‐2,4‐bis (1,1‐dimethylethyl)	Antibacterial, antifungal, antioxidant, anticancer properties. Provides protection against TMT (trimethyltin)‐induced cognitive dysfunction (Ren et al., 2019)	1.16
3	9.737	Nonadecane	Volatile oil component, Plant metabolite (Mojarrab et al., 2013)	0.95
4	10.635	Sucrose	Sweetening agent, osmolyte, antioxidant in inverted sugar form (Awadelkareem et al., 2022)	7.1
5	11.116	4‐Hydroxy‐2‐methylpyrrolidine‐2‐carboxylic acid	Antitumor activity, useful chiral building blocks for the organic synthesis of pharmaceuticals	36.6
6	11.866	Ethyl α‐D‐glucopyranoside	Skin moisturizer. Reduces hepatic function disorder, transepidermal water loss, and skin roughness following UVB irradiation (Bogaki et al., 2017).	21.9
7	12.701	n‐Hexadecanoic acid	Hypercholesterolemic, Hemolytic, Antiandrogenic, Nematicide, Antioxidant, Pesticide, 5‐α reductase inhibitor (Suja & Sivakala, 2021)	13.2
8	13.957	Oleic acid	Anti‐inflammatory, Anticancer, Antiandrogenic, Aneniagenic, Dermatitigenic, Hypercholesterolemic, Insectifuge, 5‐α reductase inhibitor (Suja & Sivakala, 2021)	9.5
9	15.107	Phenol, 3‐pentadecyl	Cytotoxic, fungicidic, and antibacterial agents (Cieślik‐Boczula et al., 2009)	3.3
10	16.325	Phenol, 3‐pentadecyl	Cytotoxic, fungicidic, and antibacterial agents (Cieślik‐Boczula et al., 2009)	2.1
11	16.647	Diisooctyl phthalate	Allelopathic, insecticidal, and antimicrobial agents (Huang et al., 2021)	1.6
12	17.894	Phenol, 3‐pentadecyl	Cytotoxic, fungicidic, and antibacterial agents (Cieślik‐Boczula et al., 2009)	1.2

**TABLE 2:** Phytochemicals identified in ethanolic extract of *Cassia angustifolia* leaves through GC–MS analysis.

Sr. No.	Retention time	Compound name	Function	Area%
1	7.019	1‐Butanol, 3‐methyl‐, formate	Aromatic and flavoring agent with antimicrobial properties (Nair & Gangaprasad, 2017)	12.8
2	11.187	D‐Glycero‐L‐gluco‐heptose	Antibacterial and antipathogenic properties (Kadhim et al., 2016)	4.9
3	11.939	L‐Glucose	Cancer cells detection, visualization, and characterization by L‐glucose fluorescent probes (Anastasiou et al., 2021), Laxative, Colon‐cleansing agent (Raymer et al., 2003)	16.05
4	12.426	3‐O‐Methyl‐D‐glucose	Nonmetabolizable glucose analog used as a marker for glucose transport (McWhorter et al., 2010)	30.8
5	12.687	n‐Hexadecanoic acid	Hypercholesterolemic, Hemolytic, Antiandrogenic, Nematicide, Antioxidant, Pesticide, and 5‐α reductase inhibitor (Liang et al., 2016)	17.4
6	13.453	Phytol	Anti‐inflammatory, antimicrobial, and anticancer agents (Nair & Gangaprasad, 2017)	5.2
7	14.007	9,12‐Octadecadienoic acid (Z,Z)‐	Anti‐inflammatory, Anticancer, Antiarthritic, Antioxidant Antiandrogenic, Antiacne, Antieczemic, Anticoronary, Hypercholesterolemic, Hepatoprotective, Antihistaminic, Nematicide, Insectifuge, 5‐α reductase inhibitor (Liang et al., 2016)	6.5
8	14.146	9,12,15‐Octadecatrienoic acid (Z,Z,Z)‐	Antibacterial, antioxidant, antiatherosclerotic, and anti‐inflammatory properties. Prevents coronary diseases and hyperkeratinization (Liang et al., 2016)	5.2

Plants have the potential to be used as medicines due to the ability to synthesize bioactive secondary metabolites (phytochemicals) and their physiological action. The medicinal effects of a plant depend upon the nature of the phytochemicals present in it. Some of these chemical compounds have therapeutic potential and can treat various illnesses, including cancer (Bakhshi & Bagherzade, 2021 #93). Specifically, the compounds containing carboxylic functional groups have been reported to possess anticancer and antibacterial properties (Jha & Prasad, 2010).

The phytochemical analysis of pistachio nuts and senna leaf extracts reported the presence of phenols, polyphenols, alkaloids, terpenoids, glycosides, fatty acids, phthalate esters, and carboxylic acid esters that may be responsible for antioxidant, antibacterial, anti‐inflammatory, and anticancer activity of the AgNPs synthesized from them (Osman et al., 2024 #96).

### 3.2 Synthesis of silver nanoparticles

Only the aqueous extracts were utilized to prepare silver nanoparticles. These extracts may have acted as the reducing as well as the stabilizing agents during AgNPs synthesis (Kumar & Yadav, 2009). When the plant extracts were added to AgNO_3_ solution, the mixture turned from colorless to dark brown. This color shift demonstrated that silver ions (Ag^+1^) present in the AgNO_3_ solution have been reduced to silver metal (Ag^0^). The synthesized AgNPs were dark brown with a silver sheen. Similar results had previously been reported by Sastry et al. (2003). They discovered that AgNPs exhibit striking colors, from light yellow to brown. Shankar et al. (2003) noted that AgNPs display a yellowish‐brown color in aqueous solution due to excitation of SP vibrations in metallic nanoparticles. Mittal et al. (2013) reported that the temperature, pH, source and concentration of plant extract, metal salt concentration, and the incubation time of precursor salt solution and plant extract influence the green synthesis of nanoparticles.

The bioactive phytocompounds of senna are anthraquinone derivatives including phytosterols, chrysophanol, kaempferol, sennosides, emodin, aloe‐emodin, and rhein. These are reported to be actively involved in the formation of AgNPs (Amaladhas et al., 2012). Furthermore, the leaves of *C. angustifolia* contain abundant polyphenols and flavonoids. According to He et al. (2017), these phytochemicals' ‐OH groups work as potent stabilizing and reducing agents. So, they might be implicated in the formation of AgNPs.

In 2011, Bolling et al. mentioned that the *Pistacia vera* nuts are rich in phenolic compounds, polyphenols, flavonoids, isoflavones, anthocyanins, proanthocyanidins, phytosterols, stilbenes, carotenoids, and phytates. Pistachios also contain resveratrol, γ‐tocopherol, isoquercetin tocopherols, terpenoids, and gallic acid (Mandalari et al., 2021). These phytocompounds supposedly contribute to the reduction of complex metal salts to metal ions.

### 3.3 Characterization of silver nanoparticles

Ultraviolet–visible spectroscopy analyzes the light extinction (absorbance and scattering) through a substance in the ultraviolet–visible range (170–780 nm). It is a useful technique for studying, identifying, and characterizing nanomaterials. Specific functional groups in NPs interact with particular light wavelengths to produce distinct optical properties that depend on the nanoparticle surface's refractive index, concentration, size, shape, and aggregation state. The FTIR spectrums of AgNPs were studied to identify the functional groups adsorbed on the nanoparticles' surface in order to determine the potential biomolecules responsible for the silver ions (Ag^+1^) reduction and capping of the bio‐reduced NPs, while high‐resolution SEM examined the shape and surface morphology of NPs.

#### 3.3.1 Ultraviolet–visible spectroscopy

**FIGURE 4:**
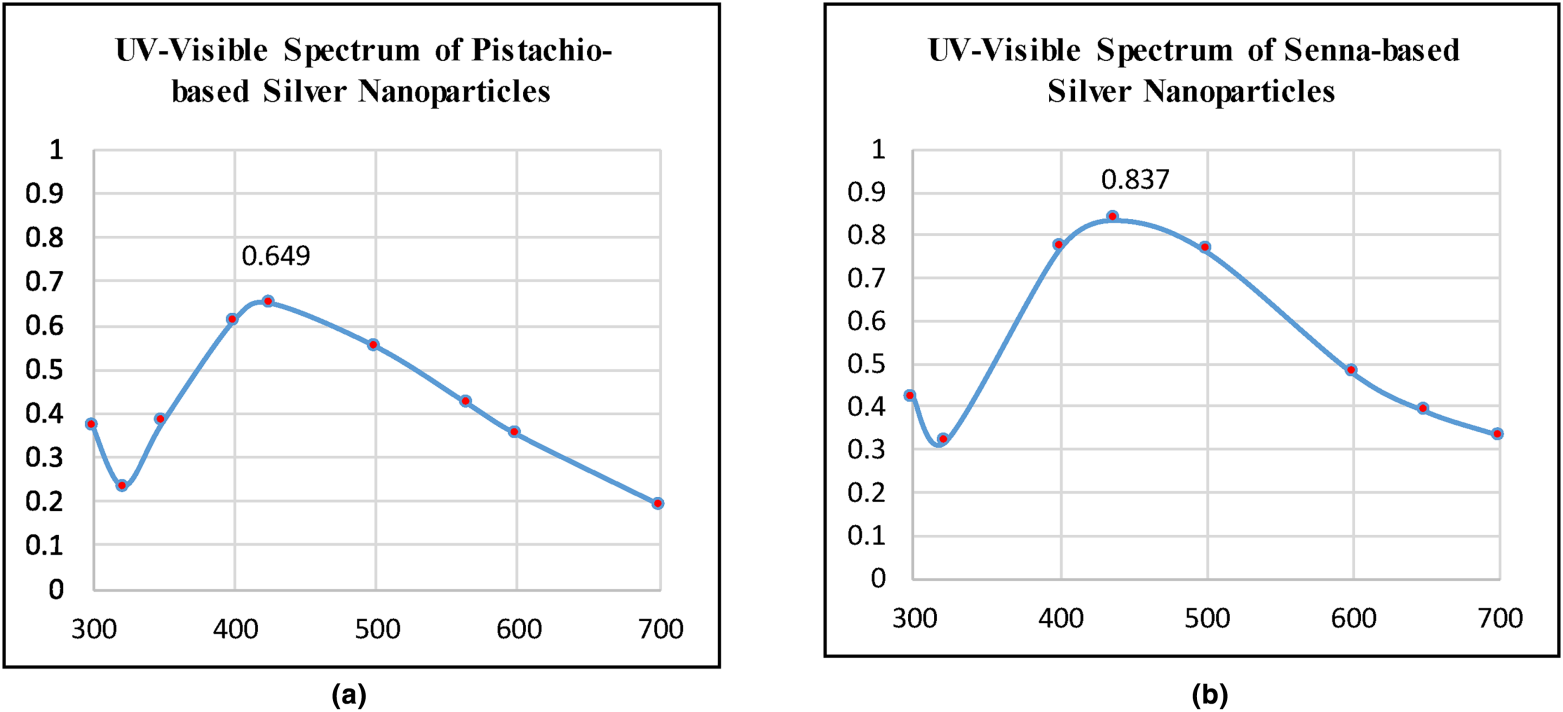
(a, b) Ultraviolet–visible spectrum of pistachio and senna‐derived AgNPs showing peak absorbance (OD = 0.649A^o^, OD = 0.837A^o^) at 425 nm and 437.5 nm, respectively, confirming nanoparticle synthesis.

Ultraviolet–visible spectroscopy is the extensively used method for describing the nanoparticles structurally. The formation of nanoparticles is noted by the changing color of the solution which is caused due to the excitation of surface plasmon vibrations of AgNPs (Bakhshi & Bagherzade, 2021). The C–O bond stretching and shift in vibrations of the C–H, O–H, and N–H bonds during NP synthesis indicate the occurrence of reduction process. Ultraviolet–visible and FTIR analyses are used to evaluate these critical alterations in the structure of chemical bonds. As illustrated in Figure 4a,b, the ultraviolet–visible spectrums of senna and pistachio AgNPs displayed the peak absorbance at 437.5 nm (OD = 0.837A^o^) and 425 nm (OD = 0.649A^o^), respectively. This confirms the reduction of silver ions by using phytoextracts and the production of stable nanoparticles. The broadened peaks indicate the synthesis of polydispersed large NPs because of slow reduction rates (Golabiazar et al., 2019).

When metallic nanoparticles get exposed to ultraviolet–visible light, they exhibit the SPR phenomenon due to simultaneous oscillations of free electron in resonance with the light wave's frequency. As a result, the SPR absorption bands form in the visible region of the ultraviolet–visible spectrum. This SPR stimulation is also responsible for the yellowish‐brown color of AgNPs in aqueous solution. Yakout and Mostafa (2015) reported that the AgNPs synthesized from *Spirulina Platensis* had SPR band in 400–480 nm range and its intensity increased linearly with contact time of reducing agent and the metal salt.

#### 3.3.2 Fourier‐Transform Infrared Spectroscopy (FTIR)

The most plausible biomolecules responsible for the reduction of silver nitrate as well as capping and stabilization of manufactured silver nanoparticles were identified using FTIR spectroscopy.

**FIGURE 5:**
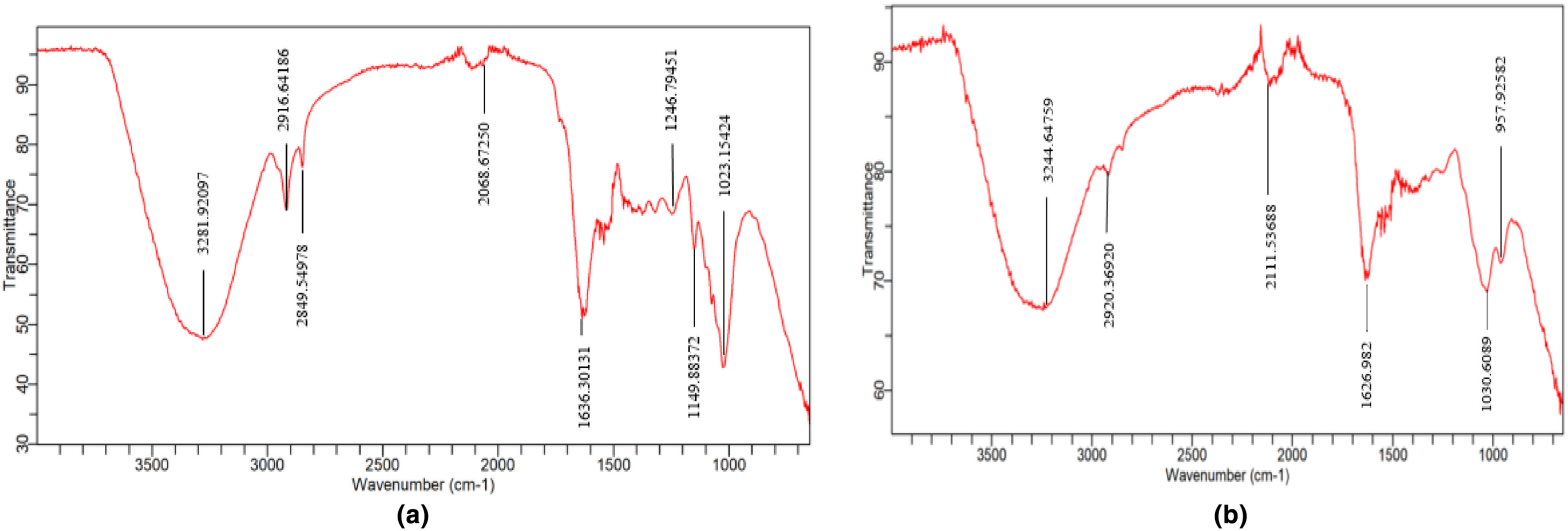
(a, b) FTIR spectrum of silver nanoparticles of *C. angustifolia* (a) and *P. vera* (*b*).

In Figure 5a, the broad IR band at 3281.92 cm^−1^ is formed due to O–H stretching corresponding to high alcohol and phenol concentration (Patil et al., 2020). The peak at 2916.64 cm^−1^ shows O–H stretch in carboxylic acid (hexadecanoic acid) (Khyade et al., 2015), while the transmittance from 2849.54 cm^−1^ to 2068.67 cm^−1^ is assigned to C–H stretch (Kavaz et al., 2018) and C ≡ C stretching in alkyne, respectively. The C–H stretching confirms the presence of flavonoid pigments. O–H of carboxylic acids in sennosides or C=O group in flavone derivatives formed the peak at 1636.30 cm^−1^ (Amaladhas et al., 2012). The transmittance from 1246.79 cm^−1^ to 1149.88 cm^−1^ represents the C‐N stretching due to aliphatic amines and C=O stretching in alcohol or aliphatic ether. C‐N and C=O stretch are typically found in aliphatic amines, which are constituents of proteins involved in metal ion reduction and stabilization. Mohanta et al. (2016) speculated that hydroxyl and carbonyl groups are majorly involved in AgNPs synthesis. The peak at 1023.15 cm^−1^ is because of –C–O (Amaladhas et al., 2012).

In Figure 5b, the band at 3244.64 cm^−1^ belongs to O–H stretching due to high alcohol, phenol (phenol, 3‐pentadecylphenol‐2,4‐bis (1,1‐dimethylethyl)), and carboxylic acid (4‐hydroxy‐2‐methylpyrrolidine‐2‐carboxylic acid, n‐Hexadecanoic acid) concentration (Kavaz et al., 2018). A peak at 2920.36 cm^−1^ represents C–H bond stretching in alkanes (Golabiazar et al., 2019), while the one at 2111.53 cm^−1^ shows weak C ≡ C stretching in alkyne. An aromatic group showing transmittance at 1626.98 cm^−1^ is responsible for C=C stretching. A peak at 1030.60 cm‐^1^ is attributable to the C–O–C bending mode and the band at 957.92 cm^−1^ represents trans = C–H out‐of‐plane bending (Golabiazar et al., 2019).

Figure 5a,b shows that Ag^+1^ ions are bio‐reduced to AgNPs by *C. angustifolia* and *P. vera* extracts, respectively. This could be verified through the C–H, O–H, N–H, C–N, and C–O bond stretching and vibrations (Golabiazar et al., 2019). Probably, phenols, sennosides, flavonoids, esters, aromatic and carbonyl compounds, glycosides, amines, and proteins of the source plants are involved in reduction, stabilization, and capping of AgNPs.

#### 3.3.3 Scanning electron microscopy (SEM)

**FIGURE 6:**
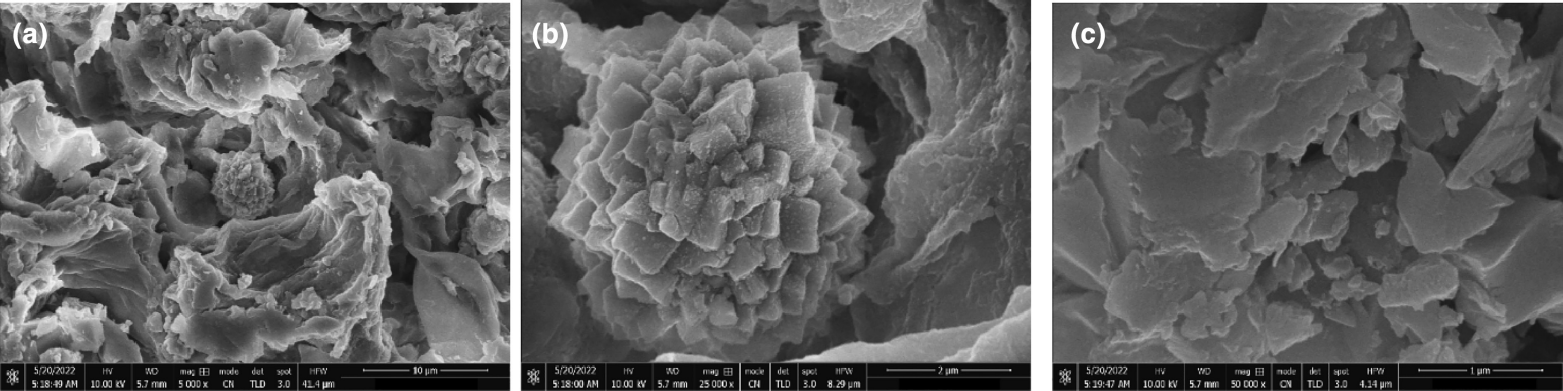
SEM images of biosynthesized senna–AgNPs.

**FIGURE 7:**
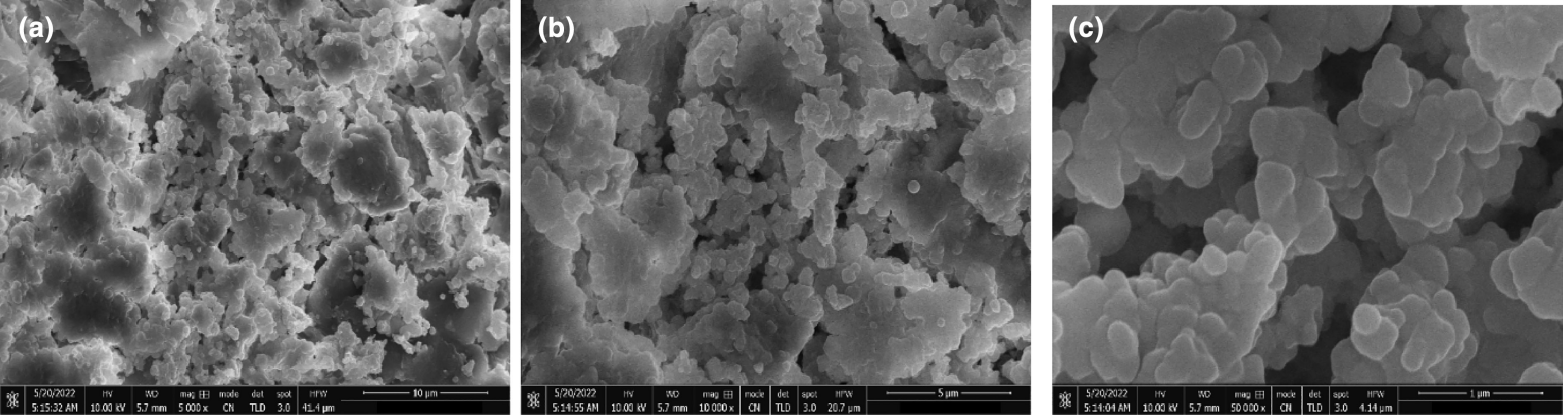
SEM images of biosynthesized pistachio–AgNPs.

Figures 6 and 7 show SEM images of silver nanoparticles at 300 and 1200 magnifications. The AgNPs of *C. angustifolia* have asymmetrical shapes and are aggregated into large flower and cloud‐like structures that lack a clearly defined morphology. They are polydispersed.

*P. vera*‐derived AgNPs are mostly spherical or ellipsoidal in shape, while some display an irregular morphology. These AgNPs are highly agglomerated and polydispersed.

The agglomeration of AgNPs could be due to the induced dehydration or deposition processes during sample preparation for scanning electron micrograph. The amount of the reducing agent and other reaction parameters affect how much agglomeration occurs (Kanwal et al., 2019).

### 3.4 Evaluation of antibacterial activity

#### 3.4.1 Disk diffusion assay

**FIGURE 8:**
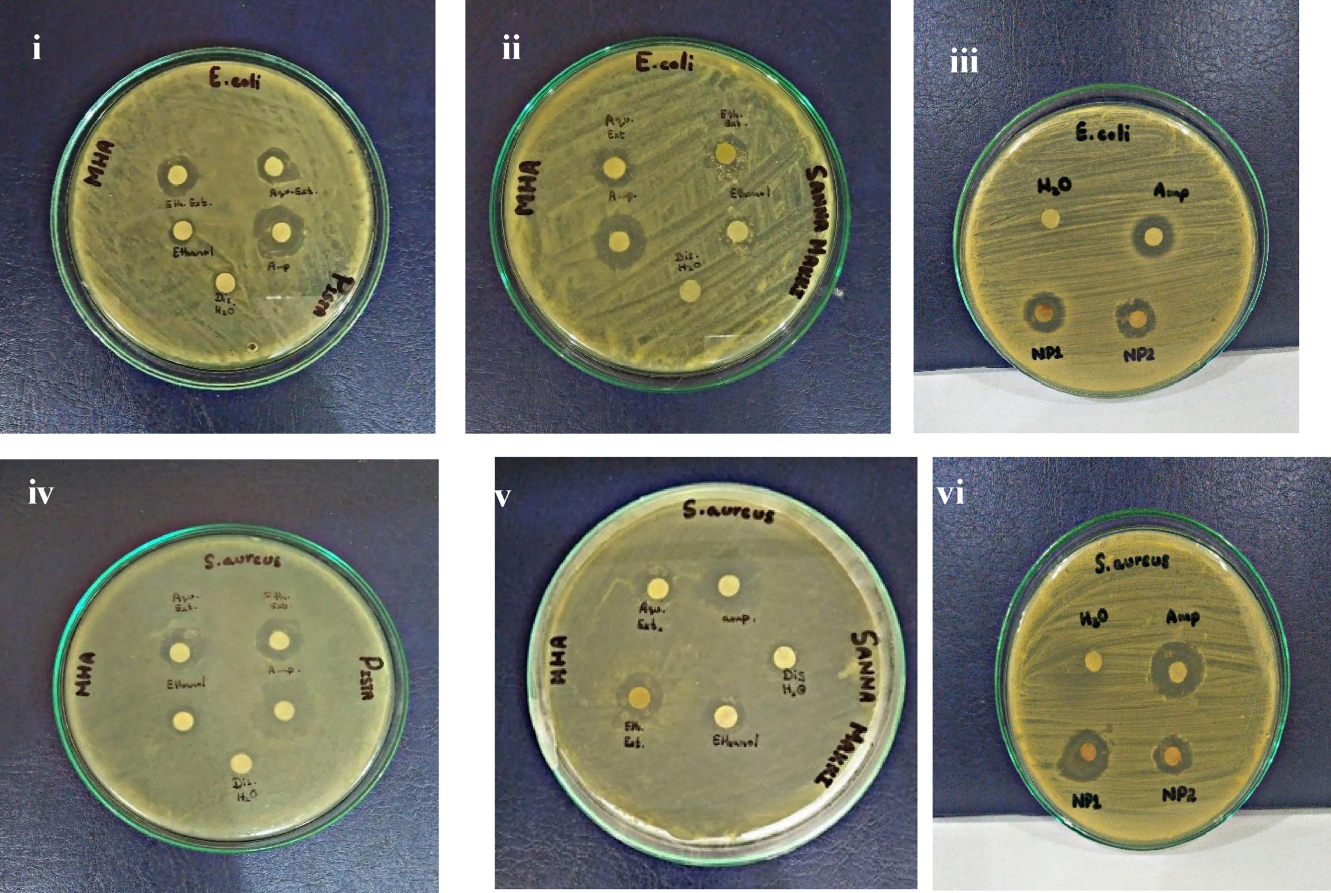
Study of antibacterial potential of pistachio extracts (i. *E. coli*, ii. *S. aureus*), senna extracts (iii. *E. coli*, iv. *S. aureus*), pistachio–AgNPs1, and Senna–AgNPs2 (v. *E. coli*, vi. *S. aureus*).

Kirby–Bauer assay was used to assess the antibacterial properties of plant extracts and AgNPs against *S. aureus* and *E. coli*. Ampicillin demonstrated the greatest antibacterial activity in all cases, forming large ZOIs. Overall, aqueous extracts outperformed ethanolic extracts. Ampicillin, aqueous extracts, and AgNPs were more potent against *S. aureus*. Distilled water had no effect on the microbial growth, ethanol generated a small zone (Table 3, Figure 8). To accurately assess antibacterial activity, the value of ethanol's ZOI was subtracted from that of ethanolic extracts.

**TABLE 3:** Comparison of ZOI of test samples with ampicillin (positive control) and distilled water (negative control) against *E. coli* and *S. aureus*.

Sample type	Zone of inhibition (mm)		ZOI (mm) after subtracting disk size	
	*E. coli*	*S. aureus*	*E. coli*	*S. aureus*
Ampicillin (Positive Control)	16.3	18.6	11.3	13.6
Ethanol	5.1	5.4	0.1	0.4
Distilled water (Negative Control)	—	—	—	—
Aqueous Pistachio extract	10	14	5	9
Aqueous Senna extract	11.9	12.6	6.9	7.6
Ethanolic Pistachio extract	8.4	10.7	3.4	5.7
Ethanolic Senna extract	9.6	7.5	4.6	2.5
Pistachio–AgNPs (NP1)	13	16.2	8	11.2
Senna–AgNPs (NP2)	12	12.5	7	7.5

*Note*: The values of ampicillin and ethanol represent the mean of three and two determinations. The size of the used disks was 5 mm.

Bisignano et al. (2013) reported that raw pistachio extracts are more effective for Gram‐positive bacteria than Gram‐negative strains, as evidenced by large ZOI, lower MIC, and MBC values. According to Ahmed et al. (2016), *C. angustifolia* is packed with flavonoids that contribute to its antimicrobial response. In 2012, Amaladhas et al., reported similar results for *C. angustifolia*‐mediated AgNPs.

#### 3.4.2 Determination of minimum inhibitory concentration

The MIC of an antibacterial agent is its minimum concentration that generates bacteriostatic action by visibly repressing bacterial growth. In the present study, the MIC values for test samples ranged from 0.3125 mg/mL to about 5 mg/mL. The ethanolic extracts of *C. angustifolia* and *P. vera* had the lowest MIC (0.3125 mg/mL) against *E. coli* and *S. aureus*, respectively. Aqueous *P. vera* extract exhibited the highest MIC (5 mg/mL) against both strains. *C. angustifolia* extracts inhibited bacterial growth more effectively than *P. vera* extracts. Overall, the nanoparticles outperformed the plant extracts (Table 4).

**TABLE 4:** Comparison of MIC and MBC of test samples.

Sample type	MIC (mg/mL)		MBC (mg/mL)	
	*E. coli*	*S. aureus*	*E. coli*	*S. aureus*
Aqueous Pistachio extract	5	5	10	5
Aqueous Senna extract	2.5	0.625	2.5	1.25
Ethanolic Pistachio extract	1.25	0.3125	2.5	5
Ethanolic Senna extract	0.3125	0.625	1.25	2.5
Pistachio–AgNPs (NP1)	1.25	1.25	2.5	2.5
Senna–AgNPs (NP2)	0.625	0.625	1.25	1.25

*Note*: The values represent the mean of three determinations.

The antibacterial activity of all test samples against both strains was dose dependent. They were substantially more potent against *S. aureus* than *E. coli*. These outcomes indicate that *S. aureus* is considerably more sensitive to bacteriostatic agents than *E. coli*. Similar results were reported by Pirtarighat et al. (2019). Sood et al. (2012) previously demonstrated that *C. angustifolia* leaves exhibit maximum antibacterial potency against *E. coli*, *S. aureus*, and *B. subtilis*.

AgNPs displayed identical bacteriostatic potential against the bacterial strains. These findings concur with those published by Kim et al. (2011). In 2020, Parvekar et al. also reported similar inhibitory concentrations for AgNPs of *C. angustifolia*. The precise mechanism of bacteriostatic action of nanoparticles remains still unknown. However, it is theorized that they release metal cations which attach to the negatively charged bacterial cell wall and interact with functional groups causing protein denaturation and ultimately cell death. NPs can potentially deface cell membranes by increasing protein leakage. ROS generation has also been linked to the antibacterial activity of NPs (Sathishkumar et al., 2010).

#### 3.4.3 Assessment of minimum bactericidal concentration

MBC is the minimal antimicrobial agent concentration required to eradicate 99.9% of bacteria in a 24‐h incubation period under controlled environmental conditions. It assesses the bactericidal potency of antimicrobials. Also known as MLC (minimum lethal concentration), it can be calculated by subculturing broth dilutions that do not show detectable microbial growth during the MIC assay.

MBC values for the test samples ranged from 1.25 to 10 mg/mL. Ethanolic extracts and AgNPs of *C. angustifolia* exhibited the smallest MBC (1.25 mg/mL) against *E. coli* and *S. aureus*, respectively. Aqueous *P. vera* extract displayed the highest MBC (10 mg/mL) for *E. coli*. Overall, the bactericidal activity of NPs was comparable to that of crude extracts from both plants (Table 4). There were no discernible differences in either the activity of *C. angustifolia* and *P. vera* AgNPs.

Pistachios are abundant in polyphenolic compounds (gallic acids and catechin) and flavonols (isoquercetin). All these phytochemicals have excellent bactericidal and bacteriostatic potency via cell expansion and, subsequent membrane rupture (Bisignano et al., 2013). In *C. angustifolia* extracts, flavonoids may cause the bactericidal and bacteriostatic effect (Das et al., 2010).

Ayala‐Núñez et al. (2009) reported that the MIC and MBC of Sigma‐Aldrich's AgNPs against 1 × 10^5^ CFU/mL *S. aureus* inoculum are 1.35 mg/mL and 2.7 mg/mL, respectively. The antimicrobial potency of AgNPs is inversely proportional to the inoculum concentration. Despite using a 1–2 × 10^8^ CFU/mL inoculum in the current investigation, equivalent outcomes for AgNPs were attained. The mechanisms underlying the bactericidal effect of these AgNPs may include the generation of free radicals or the binding of NPs to the bacterial cell wall as stated by Hajipour et al. (2012).

### 3.5 Inhibition of BSA denaturation assay

**FIGURE 9:**
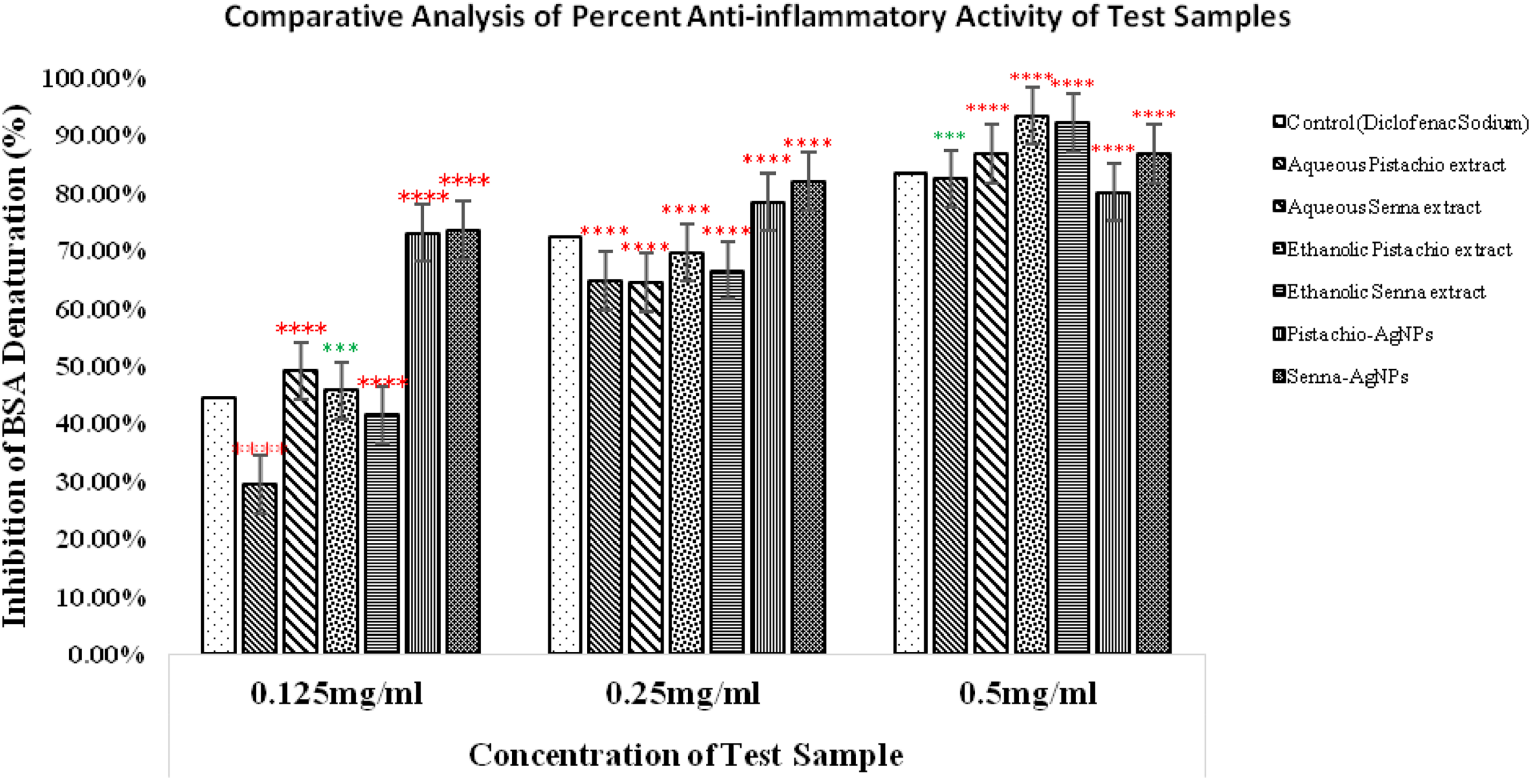
Anti‐inflammatory activity of *P. vera* and *C. angustifolia* compared with diclofenac sodium. The results are expressed as mean ± SD (*n* = 3). The statistically significant difference from the control is shown as ***p ≤ .001, ****p ≤ .0001, ^ns^ *p* > .05.

The BSA bioassay was performed to compare the anti‐inflammatory potential of plant extracts and nanoparticles to the standard drug (Diclofenac Sodium) as shown in Figure 9. In a dose‐dependent manner, all tested doses effectively reduced BSA denaturation. Diclofenac sodium displayed 44.44%, 72.22%, and 83.33% inhibition at 0.125, 0.25, and 0.5 mg/mL, respectively. Aqueous pistachio extract at 0.125 mg/mL demonstrated the lowest activity (29.38%), while the highest antidenaturation percentage was recorded for ethanolic (92.05%) and aqueous (93.30%) *C. angustifolia* extracts at 0.5 mg/mL. However, *P. vera* AgNPs and aqueous extract were less effective in inhibiting BSA denaturation than *C. angustifolia*. Nonetheless, their results are on par with those of diclofenac sodium. Other test samples outperformed the standard drug in terms of anti‐inflammatory activity.

Inflammation is the immune system's response to stimuli elicited by inflammatory factors such as microbial infections, heat, physical injuries, or chemical irritations. It is a contributing factor in the pathogenesis of stroke, arthritis, and cancer (Ricciotti & FitzGerald, 2011). Increased cytokines production and ROS significantly modulate the inflammatory response ultimately leading to protein denaturation. Protein denaturation is strongly correlated with inflammation (Mizushima, 1966). Therefore, the ability of a drug to inhibit protein denaturation indicates its anti‐inflammatory efficacy. NSAIDs are the most commonly prescribed medications for inflammatory diseases (Saso et al., 2001). But they are linked to cardiovascular problems and gastrointestinal side effects. Phytocompounds, particularly antioxidants, have been identified as potential anti‐inflammatory agents (Osman et al., 2016) as they can minimize the oxidative damage caused to proteins.

Raw pistachios suppresses tissue damage, cytokine TNF‐α and IL1β synthesis, and modulates various anti‐inflammatory responses in hyperglycemic rats (Di Paola et al., 2018). *P. vera* is rich in polyphenols with anti‐inflammatory properties and is used to treat colitis and paw edema. Inflammation is diminished by pista and its derivatives by either direct or indirect mechanisms. Cuellar et al. (2001) reported the topical anti‐inflammatory action of *C. angustifolia* extracts. In 2013, Singh et al. reported that phenolic compounds, anthraquinones, flavonoids, and naphthopyrone glycosides in *Cassia* species contribute to their anti‐inflammatory properties.

### 3.6 DPPH (2,2‐Diphenyl‐1‐picrylhydrazyl) antioxidant assay

Cellular respiration, metabolic processes, and environmental oxidants generate several potentially harmful by‐products in the body. One of them is called ROS (reactive oxygen species). However, ROS plays an important role in cell signaling. They have the potential to cause oxidative stress in cells. ROS are highly active as molecules, ions, and free radicals because they contain unpaired electrons (Poljsak et al., 2013; Riaz et al., 2016). Their excessive presence in the human body may cause certain ailments like cardiovascular disease, tumors, cancers, diabetes, cataract, or aging. Because of their excellent reducing power, antioxidants are essential for scavenging free radicals from the body (Kauser et al., 2018).

**FIGURE 10:**
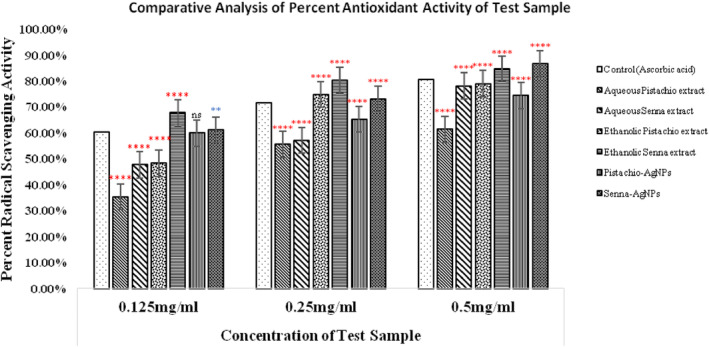
Comparison of radical scavenging activity of phytoextracts and silver nanoparticles of *P. vera* and *C. angustifolia* with ascorbic acid. The results are expressed as mean ± SD (*n* = 3). The statistically significant difference from the control is shown as **p ≤ .01, ****p ≤ .0001, ^ns^ *p* > .05.

Therefore, DPPH assay was performed to assess the antioxidant potential of *P. vera* and *C. angustifolia* phytoextracts as well as AgNPs (Figure 10). In a dose‐dependent manner, the test samples significantly terminated DPPH free radicals present in the mixture at all tested concentrations. Figure 10 shows that increasing sample concentration upsurges the reducing power of a test sample. Ascorbic acid (positive control) exhibited scavenging activity of 60.19 ± 0.017%, 71.57 ± 0.35%, and 80.65 ± 0.43% at 0.125, 0.25, and 0.5 mg/mL concentration, respectively. Of all the test samples, aqueous pistachio extract displayed the lowest potency, while the ethanolic extract (84.6 ± 0.35%) and AgNPs (86.57 ± 0.22%) of senna leaves were found to have the highest antioxidant percentages when used at 0.5 mg/mL. Generally, ethanolic extracts demonstrated better antioxidant action than the aqueous extracts. This is due to the poor extraction of phenols and flavonoids in aqueous solvents.

Pistachios' antioxidant potential is attributed to the phenolic compounds like gallic acid, cyanidin‐3‐O‐galactoside, rutin, catechin, epicatechin, and eriodictyol‐7‐O‐glucoside (Sonmezdag et al., 2017). Antioxidant substances containing phenolic compounds neutralize DPPH radicals through their radical scavenging ability or hydrogen‐donating capacity. The presence of an ortho‐dihydroxy structure and the molecular planarity of phenolic compounds make them efficient hydrogen donors. Hence, a strong correlation between total phenolic content and antioxidant activity has been reported by Taghizadeh et al. (2018). In 2021, Elhadef et al. stated that the solvent's polarity increases the DPPH radical scavenging activity.

Senna contains flavonoids rutin, scutellarein, and quercimeritrin that prevent oxidative stress. They are all powerful antioxidants due to the presence of 1,2‐dihydroxybenzene groups that are easily oxidized to orthoquinones (Ahmed et al., 2016). Senna also contains phenolic compounds and proanthocyanidins.

Two factors may be responsible for AgNPs' antioxidant potential: (i) presence of large quantities of antioxidants on their surface because of large surface area, (ii) phytochemicals that serve as capping agents (e.g., flavonoids). In 2020, Keshari et al. confirmed that AgNPs exhibit stronger antioxidant action than Vitamin C (ascorbic acid).

### 3.7 MTT anticancer assay

Following MTT assay, cell viability analysis was done using Prism GraphPad. It is shown that growth inhibition was increased with increasing concentrations of the test samples. Each group showed a significant decrease in cell viability or increase in growth inhibition when treated with the test samples under discussion.

**FIGURE 11:**
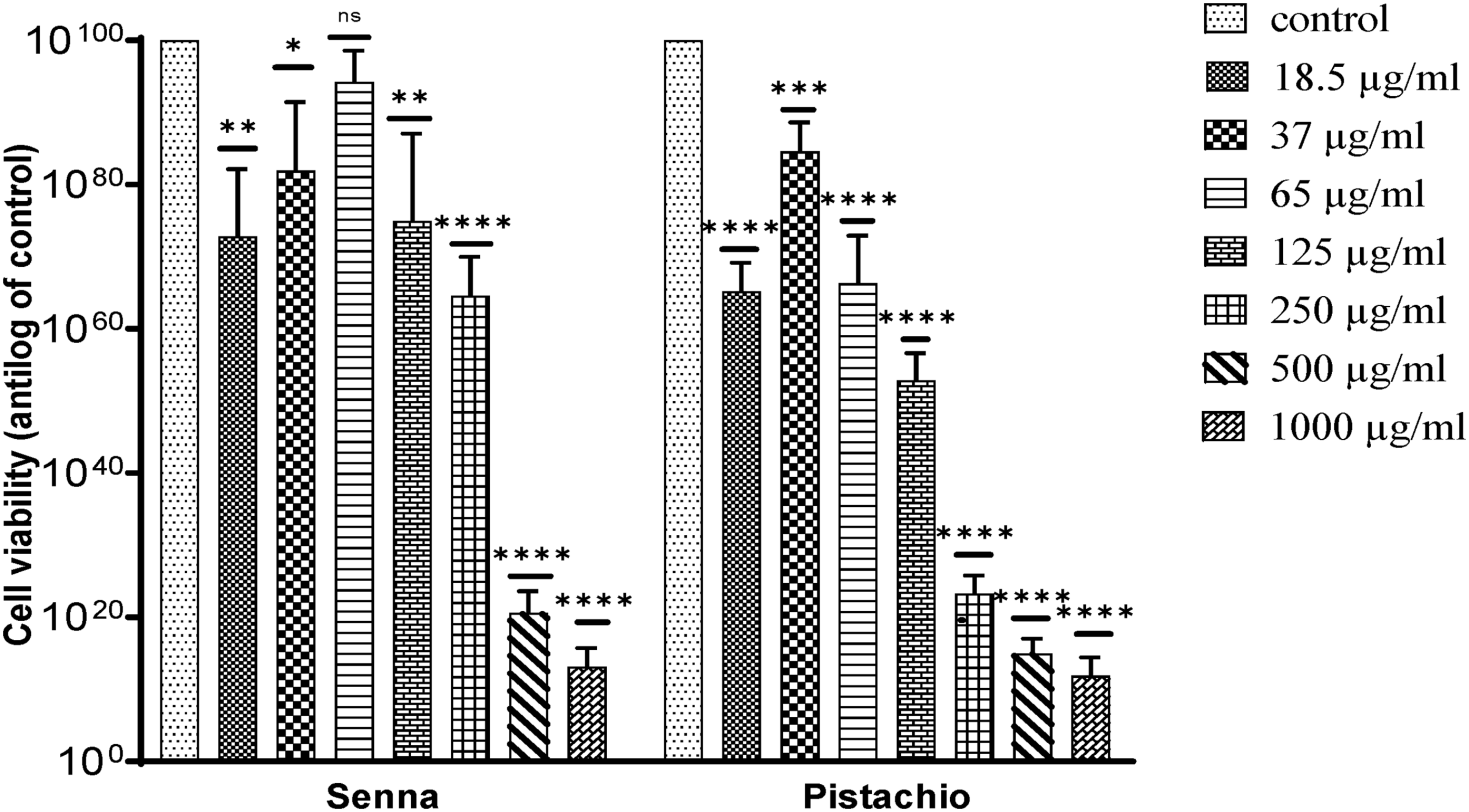
Comparative effect of phytoextracts of senna and pistachio on HepG2 cells. The data are represented as mean ± SD (*n* = 3). The statistically significant difference as compared to the control is represented as *p ≤ .05, **p ≤ .01, ***p ≤ .001, ****p ≤ .0001, ^ns^ *p* ≥ .05.

The aqueous extracts of *P. vera* and *C. angustifolia* did not show any cytotoxic effect on the HepG2 cells (Figure 11). Control (untreated cells) showed 100 ± 00% viability. The cell viability for ethanolic *C. angustifolia* extract at 18.5, 37, 65, 125, 250, 500, and 1000 μg/mL was 72.84 ± 9.23%, 81.95 ± 9.46%, 94.25 ± 4.32%, 74.94 ± 12.12%, 64.59 ± 5.39%, 20.59 ± 3.03%, and 13.17 ± 2.57%, respectively. For *P. vera* ethanolic extract, the results of cell viability obtained were 65.18 ± 3.98%, 84.6 ± 4.04%, 66.31 ± 6.59%, 52.87 ± 3.76%, 23.28 ± 2.39%, 15.001 ± 2.03%, and 11.89 ± 2.51% at 18.5, 37, 65, 125, 250, 500, and 1000 μg/mL, respectively.

**FIGURE 12:**
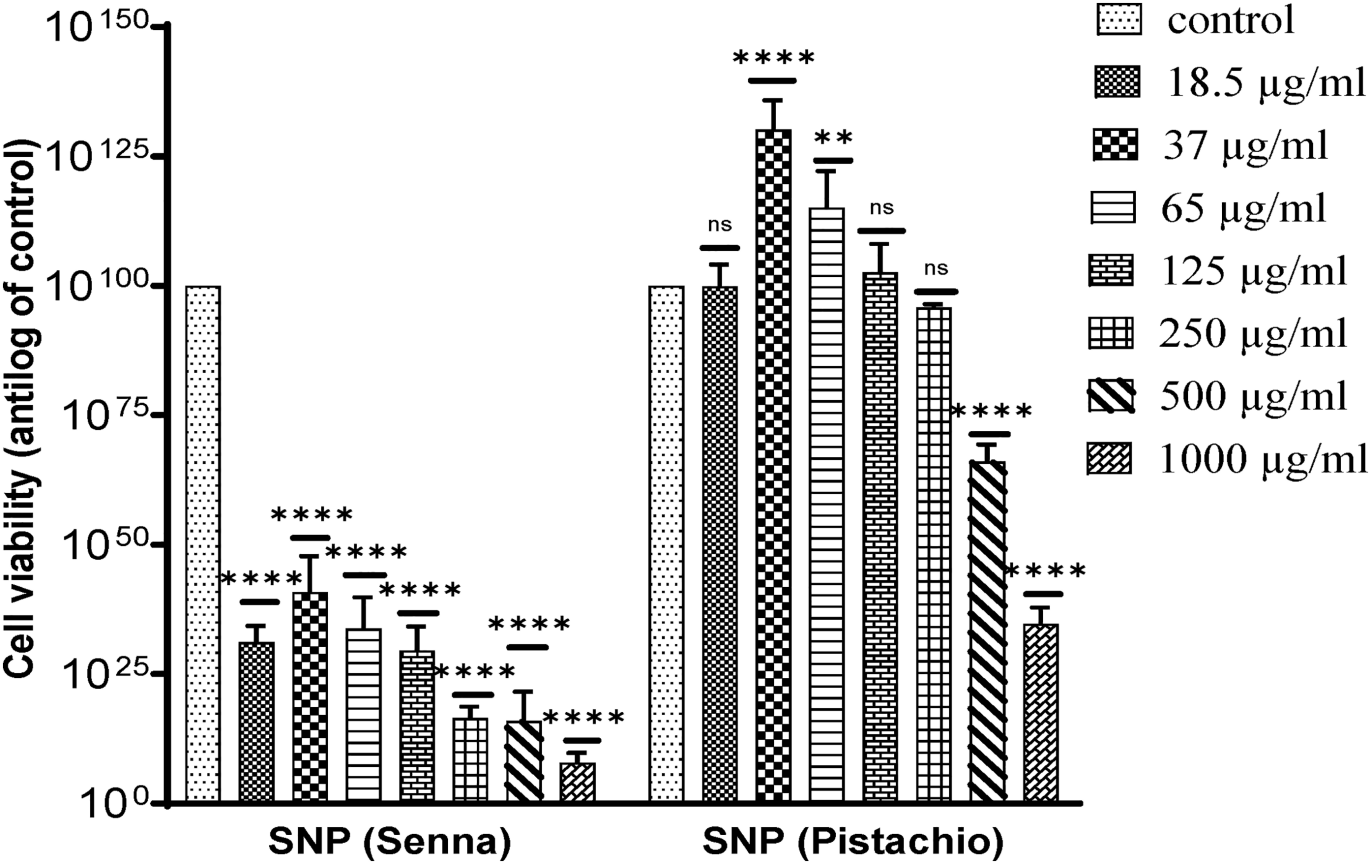
Comparative effect of silver nanoparticles (senna and pistachio) on cancer cells (HepG2). The data are represented as mean ± SD (*n* = 3). The statistically significant difference as compared to the control is represented as **p ≤ .01, ****p ≤ .0001, ^ns^ *p* ≥ .05.

**FIGURE 13:**
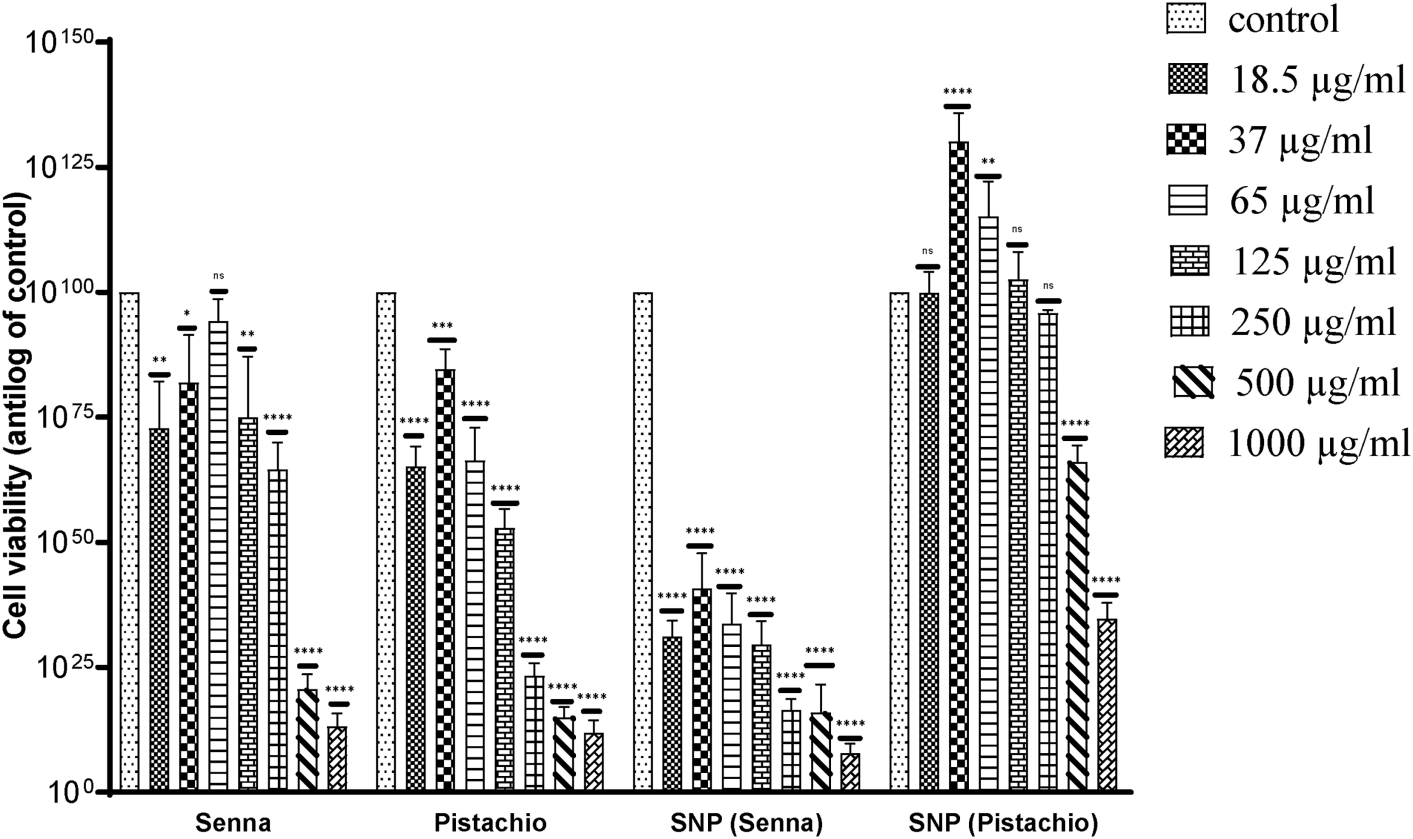
Comparative effect of phytoextracts and silver nanoparticles (senna and pistachio) on HepG2 cells. The data are represented as mean ± SD (*n* = 3). The statistically significant difference as compared to the control is represented as *p ≤ .05, **p ≤ .01, ***p ≤ .001, ****p ≤ .0001, ^ns^ *p* ≥ .05.

Comparative effect of silver nanoparticles (senna and pistachio) on HepG2 cells is shown in Figure 12. *C. angustifolia* AgNPs reduced the cell viability in a dose‐dependent manner as evidenced from these outcomes 18.5 μg/mL (34.45 ± 2.9%), 37 μg/mL (52.13 ± 5.06%), 65 μg/mL (33.73 ± 5.993%), 125 μg/mL (29.53 ± 4.59%), 250 μg/mL (24.85 ± 3.39%), 500 μg/mL (12.59 ± 2.39%), and 1000 μg/mL (5.9997 ± 2.03%), while the *P. vera* AgNPs showed the following results: 18.5 μg/mL (106.553 ± 2.81%), 37 μg/mL (124.80 ± 3.37%), 65 μg/mL (112.8 ± 3.49%), 125 μg/mL (99.92 ± 0.522%), 250 μg/mL (92.14 ± 0.52%), 500 μg/mL (7.02 ± 3.28%), and 1000 μg/mL (31.37 ± 2. 64%). Figure 13 shows the comparative effect of both the phytoextracts and silver nanoparticles (senna and pistachio) on cancer cells (HepG2).

Despite the American Cancer Society's report about declining cancer rates, the incidence rate of liver cancer is rising. Regardless of all attempts to discover novel medications, Sorafenib is the only effective treatment available for advanced liver cancer (Fathalizadeh et al., 2015). Therefore, it is crucial to discover new anticancer agents to combat hepatocellular carcinoma.

It has been established that *Pista* and *Cassia* species can be used as natural anticancer agents against different types of cancers. Consequently, in this study, the anticancer potential of *P. vera* and *C. angustifolia* phytoextracts and nanoparticles was evaluated through MTT assay on HepG2 cells. The aqueous extracts exhibited no cytotoxic effects. Even though the experiment was run twice, the results were similar in both cases. At all test concentrations, the average percentage of cell viability was greater than 100%. In 2016, Ahmed et al. reported similar outcomes for aqueous extract of *C. angustifolia* seeds against the HepG2 cells. Shirazi et al. (2006) reported that pistachio extract could not protect HepG2 cells, instead, it increased the AFB1‐induced cytotoxicity in certain circumstances.

Previous literature and our findings indicate that *P. vera* and *C. angustifolia*‐based ethanolic extracts as well as AgNPs could be promising anticancer agents for liver cancer. This could be due to high concentrations of phenolic compounds, terpenes, and flavonoids present in the source plants (Fathalizadeh et al., 2015; Koyuncu et al., 2018). As reported by Ahmed et al. (2016) and Harandi et al. (2018), the ethanolic extracts of senna seeds and pistachio pericarp exhibit anticancer properties.

Previously, no detailed anticancer study of AgNPs synthesized from *P. vera* nuts and *C. angustifolia* leaves on HepG2 cells has been conducted. However, researchers have discovered that the cytotoxic potential of AgNPs could be accredited to their ability to form ROS (reactive oxygen species). Intracellular macromolecules like proteins, lipids, and nucleic acids can interact directly or indirectly with ROS leading to cell death. They can also cause cell death by destroying the integrity of the cell membrane, inducing apoptosis, or instigating DNA damage (He et al., 2017).

## 4 CONCLUSION

This study presents a comparison of the pharmacological potential of crude extracts and silver nanoparticles produced from *Cassia angustifolia* (senna) leaves and *Pistacia vera* (pistachio) nuts. Results confirmed that the tested plant extract possesses a variety of bioactive compounds with various biological activities and are therapeutically effective. The outcomes indicated that both plants could be promising resource materials in biopharmaceutical industries. To our knowledge, this is the first research study to report the synthesis of silver nanoparticles from *P. vera* nuts.

## AUTHOR CONTRIBUTIONS

**Saba Irshad:** Conceptualization (lead); formal analysis (equal); funding acquisition (lead); project administration (lead); resources (lead); supervision (lead); visualization (supporting); writing – original draft (equal); writing – review and editing (equal). **Sabahat Iftikhar:** Formal analysis (lead); investigation (equal); methodology (equal); writing – original draft (equal). **Muhammad Riaz:** Formal analysis (supporting); methodology (supporting); software (equal); validation (equal); visualization (equal); writing – original draft (supporting); writing – review and editing (lead). **Azra Mahmood:** Data curation (supporting); formal analysis (supporting); funding acquisition (supporting); investigation (equal); resources (equal); validation (equal); writing – review and editing (supporting). **Afaq Mushtaq:** Conceptualization (supporting); funding acquisition (supporting); project administration (supporting); resources (equal); validation (equal); writing – original draft (supporting). **Yasar Saleem:** Formal analysis (supporting); funding acquisition (supporting); resources (equal); validation (equal); visualization (supporting). **Rahat Shamim:** Formal analysis (supporting); methodology (supporting); software (equal); validation (equal); visualization (supporting). **Quzi Sharmin Akter:** Data curation (supporting); funding acquisition (supporting); software (supporting); validation (equal); visualization (equal); writing – review and editing (equal).

## ACKNOWLEDGEMENTS

The authors are highly thankful to the University of the Punjab for providing the facility in conducting the experimental work. Authors are also thankful to the PCSIR Lab, Lahore, for providing the laboratory facility in conducting a part of the experimental work of this study.

## FUNDING INFORMATION

No particular funding was received for this research study.

## CONFLICT OF INTEREST STATEMENT

The authors declare no conflict of interest.

## Data Availability

Data will be available from the principal corresponding author upon reasonable request.
